# Vessel Subinvolution of the Placental Implantation Site: A Case Report and Review of Supportive Literature

**DOI:** 10.7759/cureus.13472

**Published:** 2021-02-21

**Authors:** Subramaniam Ramkumar, Teikupar Kharshiing

**Affiliations:** 1 Pathology, Woodland Hospital, Shillong, IND; 2 Obstetrics and Gynaecology, Woodland Hospital, Shillong, IND

**Keywords:** uteroplacental arteries, subinvolution, postpartum hemorrhage

## Abstract

Subinvolution of the implantation site is a significant contributor to delayed postpartum hemorrhage (PPH). There is immense literature documenting the histologic features, development, and involution of the uteroplacental site; however, practice-oriented literature on subinvolution of the implantation site is sparse. In the present study, we briefly review the physiologic characteristics associated with the normal development and involution of uteroplacental arteries and the proposed pathophysiologic attributes of subinvolution. Furthermore, we engage in a comparison of the condition with preeclampsia. Herein, we report a case of postpartum vaginal bleeding that persisted for two weeks following cesarean delivery. The bleeding was nonresponsive to conservative treatment. Sonography performed revealed that a heterogeneous intrauterine vascular mass measuring 14.6 × 9.2 × 10.4 cm^ ^was present, distending the uterine cavity. The presence of retained products of conception could not be ruled out. Therefore, to further confirm the condition, the patient underwent an emergency hysterectomy. Gross examination showed an enlarged and boggy uterus with numerous dilated and ecstatic thrombosed blood vessels in the implantation site of the endomyometrium. Histologic and immunohistochemical examination of the implantation site revealed the presence of persistently patent uteroplacental arteries with variable degrees of thrombosis adjacent to normally involuted vessels. Hence, a diagnosis of placental site vessel subinvolution (VSI) was established in this case. We also reviewed the related literature to illustrate the informative histologic findings of subinvolution. Preparing the ground for diagnosing subinvolution is important as this process defines that the cause of delayed postpartum uterine bleeding is idiopathic, rather than iatrogenic.

## Introduction

Postpartum hemorrhage (PPH) is a major cause of maternal deaths worldwide due to its association with hemorrhagic shock and the tendency to develop disseminated intravascular coagulation [1,2]. The current definition of PPH based on the quantification of blood loss has several limitations, and in most postpartum mothers, PPH has always been either underdiagnosed or overdiagnosed [3,4-8].

Causes of primary PPH

Primary PPH is prevalent during the first 24 hours post-completion of the third stage of labor. The leading causes of primary PPH are as follows: acquired coagulopathy, uterine atony, trauma associated with the lower genital tract, congenital coagulopathy, and retained products of conception [9-14].

Causes of secondary PPH

Secondary PPH usually occurs between 24 hours and six weeks post-parturition. Secondary PPH has received sparse attention, probably due to its low incidence of complications, which account for only 1% of all pregnancies, and its regular association with maternal morbidity rather than mortality [15,16]. However, secondary PPH might be fatal as it occurs from one to two weeks post-delivery when many affected patients are at home; as a result, they may not be aware that the bleeding can be fatal [1,15,17,18]. The common documented causes of secondary PPH include cesarean scar dehiscence, retained products of conception, endometritis, and uncommon neoplastic causes such as choriocarcinoma. The origin of secondary PPH usually remains unidentified when a patient is conservatively managed [10,15].

A major clinical area that has garnered interest as far as PPH is concerned is vessel subinvolution (VSI) of the placental implantation site [19]. There has been a growth of interest in understanding the pathologic characteristics of VSI and how it contributes to maternal death [15], leading to an increased focus on such cases [20]. In physiologic involution, spontaneous thrombi together with fibrotic closure of the uteroplacental vessels occur during the period of normal postpartum. The events associated with the involution take the vessels back to the nongestational state. A failure to return to this state can result in a quick but impermanent reorganization of the vessel wall and can subsequently result in VSI. When this happens, intermittent vaginal or severe profuse bleeding can occur post-delivery [6], along with rapid cardiovascular collapse, which sometimes necessitates urgent hysterectomy [10,14]. Nevertheless, subinvolution of the placental site has not been fully understood due to the lack of pathologic specimens to confirm the diagnosis [9]. In this report, we provide a description of a rare type of recurrent secondary PPH caused by local VSI after an uncomplicated cesarean delivery and present a review of the available literature to support our findings [6,10]. In our case, the diagnosis was ascertained via the histologic and immunohistochemical demonstration of large, non-involuted, persistently patent, and remodeled endomyometrial arteries in the postpartum hysterectomy specimen.

## Case presentation

A 38-year-old woman (P3L3) was brought to the hospital’s emergency department due to vaginal bleeding accompanied by clinical symptoms, such as chills and lightheadedness, which had persisted for 14 days post-cesarean delivery at the end of her pregnancy, which was described as uneventful. At the time of her admission, she complained of irregular abdominal pain. Additionally, she reported frequent profuse vaginal bleeding, during which 10 pads were soaked over a 10-hour period. Physical examination revealed that her abdomen was moderately tender; bloody vaginal discharge was also noted. The patient was afebrile and had tachycardia (blood pressure: 80/50 mmHg). The initial blood test revealed leukocytosis, with a WBC count of 12,000/μL and a hemoglobin level of 7.8 g/dL. Other laboratory findings were as follows: serum β-human chorionic gonadotropin (β-HCG): 2.75 mIU/mL; prothrombin time: 15.72 seconds (normal control: 13.1 seconds; international normalized ratio: 1.19); activated partial thromboplastin time: 26.3 seconds (normal control: 30.0 seconds); bleeding time: 2.25 minutes; clotting time: 5.5 minutes; platelet count: 280,000 cells/mm^3^; total protein level: 5.5 g/dL; albumin level: 3.1 g/dL; globulin: 2.4 g/dL; and albumin:globulin ratio: 1.3. Serum glutamic-oxaloacetic transaminase (SGOT) level was 18 U/L, alkaline phosphatase level was 62 IU/L, and serum glutamic-pyruvic transaminase (SGPT) level was 10 U/L. The clinical findings also showed that the patient had a γ-glutamyl transferase level of 10 U/L, total bilirubin level of 0.3 mg/dL, conjugated bilirubin level of 0.2 mg/dL, and an unconjugated bilirubin level of 0.1 mg/dL. The patient was resuscitated with fluids and conservatively treated with four units of packed red blood cells and tranexamic acid (one amp intravenously twice per day and stat). However, bleeding persisted and the hemoglobin levels progressively decreased.

Sonography, performed using the Voluson S7 ultrasound unit (GE Healthcare, Chicago, IL), revealed that a heterogeneous mass measuring 14.6 × 9.2 × 10.4 cm was present, distending the endometrial cavity. Substantial vascularity was observed around the anterior endometrium and was more localized in the myometrial vessel along the left anterior myometrium. The findings further showed the formation of venous varix and the indication of the subinvolution of the placental site. Despite these results, the presence of retained products of conception could not be ruled out. Therefore, to further confirm the condition, the patient underwent an emergency hysterectomy.

**Figure 1:**
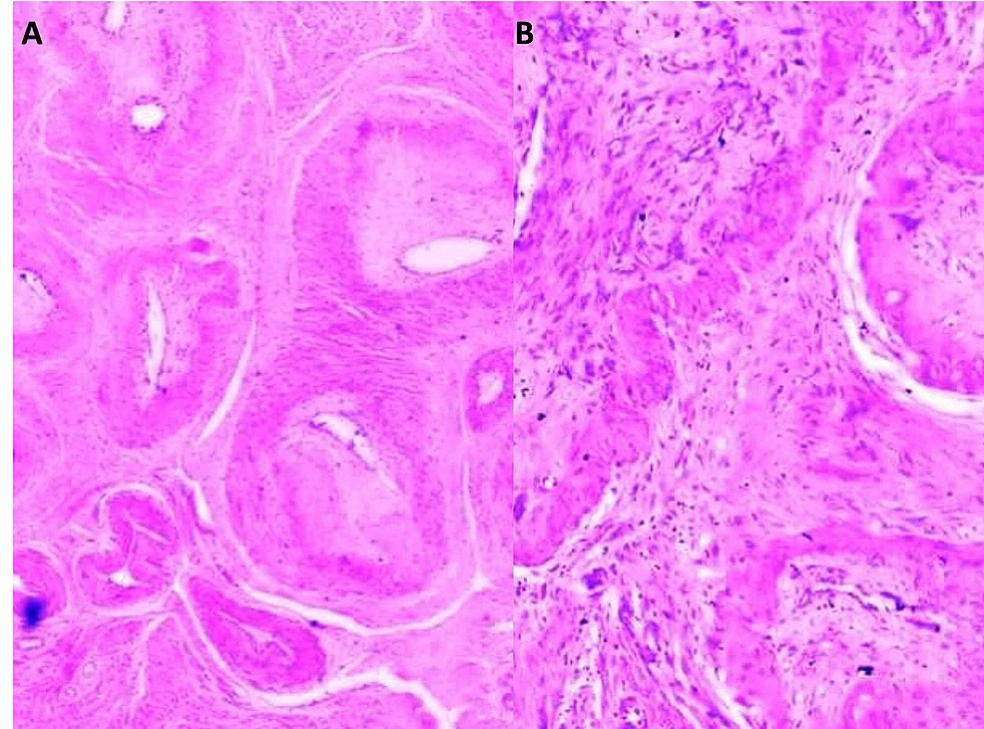
Examination findings - 1 1A: low-power view showing clusters of normally involuted uteroplacental arteries (H&E x 20). 1B: medium-power view showing fibrointimal obliteration of the uteroplacental artery lumen (H&E x 40) H&E: hematoxylin and eosin

Further assessment of the pathologic gross examination of the uterus revealed a considerable amount of thrombi in the endometrial cavity. Microscopic examination of the myometrium beneath the placental implantation site revealed the presence of persistently patent uteroplacental arteries adjacent to normally involuted vessels (Figure 1).

**Figure 2:**
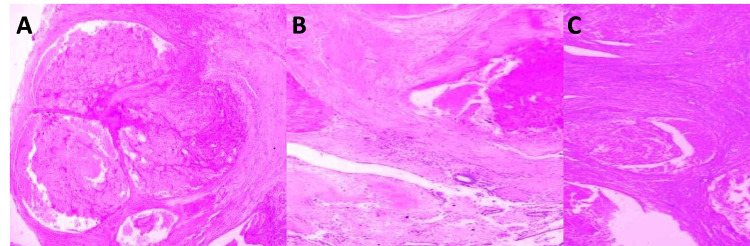
Examination findings - 2 2A-2C: low-power view of clusters of patent uteroplacental arteries filled with red cells and loose fibrin (H&E x 20) H&E: hematoxylin and eosin

Clusters of large ectatic and patent blood vessels with luminal red blood cells and loose fibrin were found in the endomyometrium (Figures 2A, 2B, 2C).

**Figure 3:**
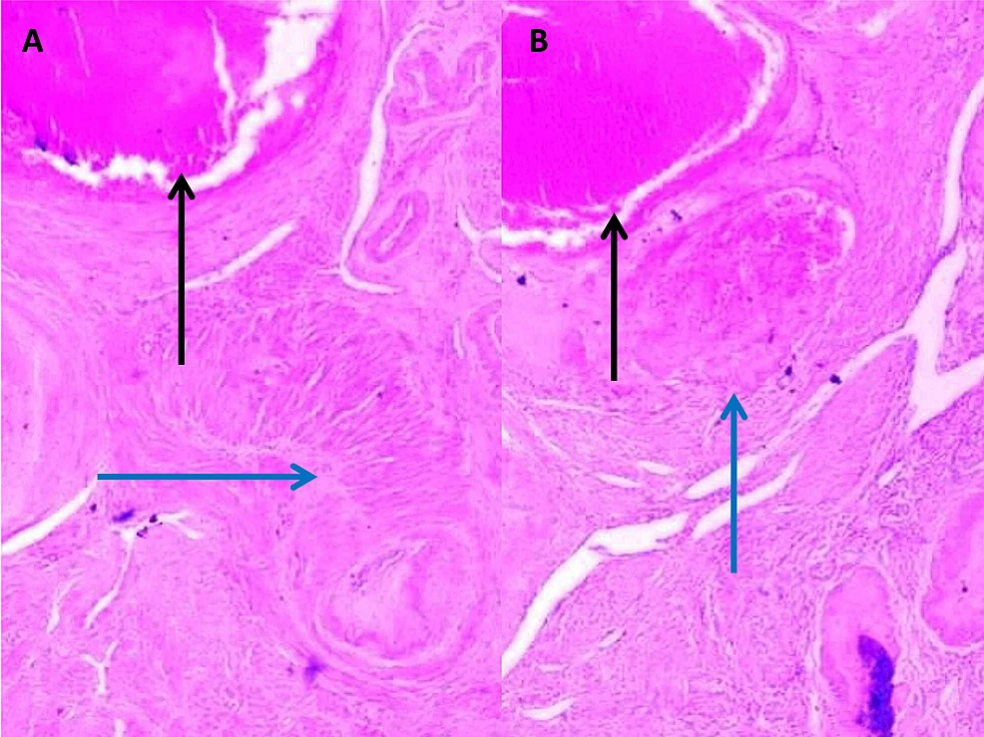
Examination findings - 3 3A-3B: low-power view of subinvoluted vessels adjacent to normally involuted vessels (H&E x 20); black arrow: subinvoluted vessel wall; blue arrow: normally involuted vessel wall H&E: hematoxylin and eosin

Numerous constant patent uteroplacental arteries with different degrees of thrombosis located next to normally involuted vessels were also observed (Figures 3A, 3B).

**Figure 4:**
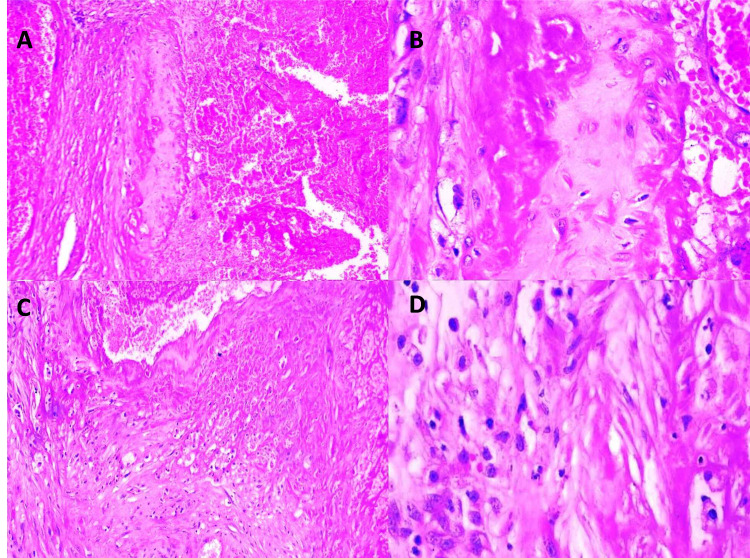
Examination findings - 4 4A-4B: persistent EVT cells within subinvoluted vessel wall (4A: H&E x 20; 4B: H&E x 40). 4C-4D: persistent EVTcells within subinvoluted interstitium (4C: H&E x 20; 4D: H&E x 40) EVT: extravillous trophoblast; H&E: hematoxylin and eosin

Persistent extravillous trophoblast (EVT) cells found within the subinvoluted vessel wall can be observed in Figures 4A, 4B, whereas EVT cells in the subinvoluted interstitium are shown in Figures 4C, 4D.

**Figure 5:**
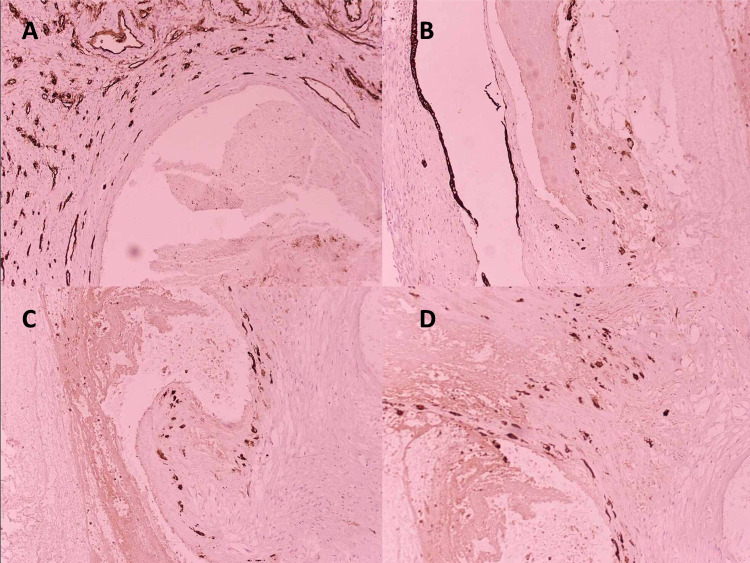
Immunohistochemical analysis 5A: subinvoluted blood vessel showing persistent EVT replacing normal endothelium; CD34 negative in the subinvoluted vessel wall; CD34 positive in the adjacent myometrial vessels (internal positive control) (x20). 5B: subinvoluted blood vessel showing numerous persistent PAN-CK-positive endovascular trophoblastic cells; PAN-CK-positive endometrial glands (internal positive control) (x20). 5C: subinvoluted blood vessel showing numerous persistent P63-positive EVT in the vessel wall (x20). 5D: subinvoluted blood vessel showing numerous persistent P63-positive EVT in the vessel wall and surrounding interstitium (x20) EVT: extravillous trophoblast

The immunohistochemical analysis determined the presence of residual trophoblasts within the abnormal vessel walls and interstitium. In the subinvoluted vessels, EVTs replaced the normal endothelium (Figure 5A), revealing multiple PAN-CK-positive EVT cells (Figure 5B) that showed P63-positive EVTs in the vessel walls as well as surrounding interstitium (Figures 5C, 5D).

**Figure 6:**
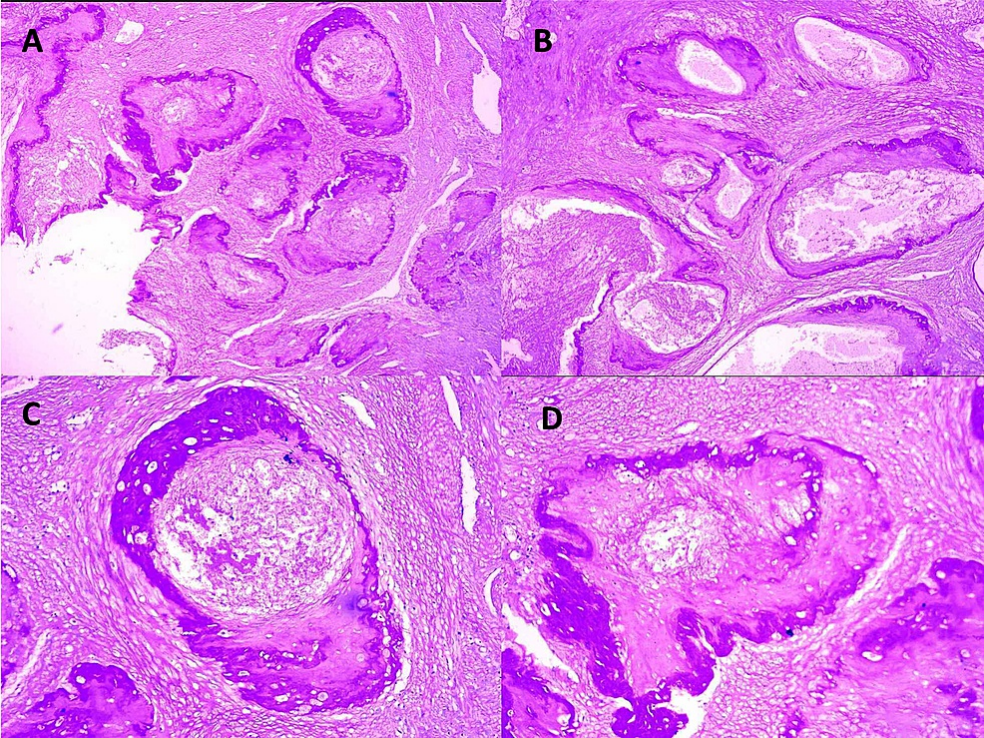
PAS stain highlighting the disrupted histomorphologic architecture of persistently patent vessels 6A, 6B: PAS stain x 20; 6C, 6D: PAS stain x 40 PAS: periodic acid-Schiff

Histochemical staining for elastin provides a significant benefit for describing the structure of the vessel wall. PAS staining was used for this purpose and revealed disrupted histomorphology of persistently patent vessels (Figures 6A, 6B, 6C, 6D).

The patient was followed up with serial beta-HCG monitoring and CBC assessment. She recovered fully and was found to be asymptomatic on follow-up.

## Discussion

Physiology of involution

**Figure 7:**
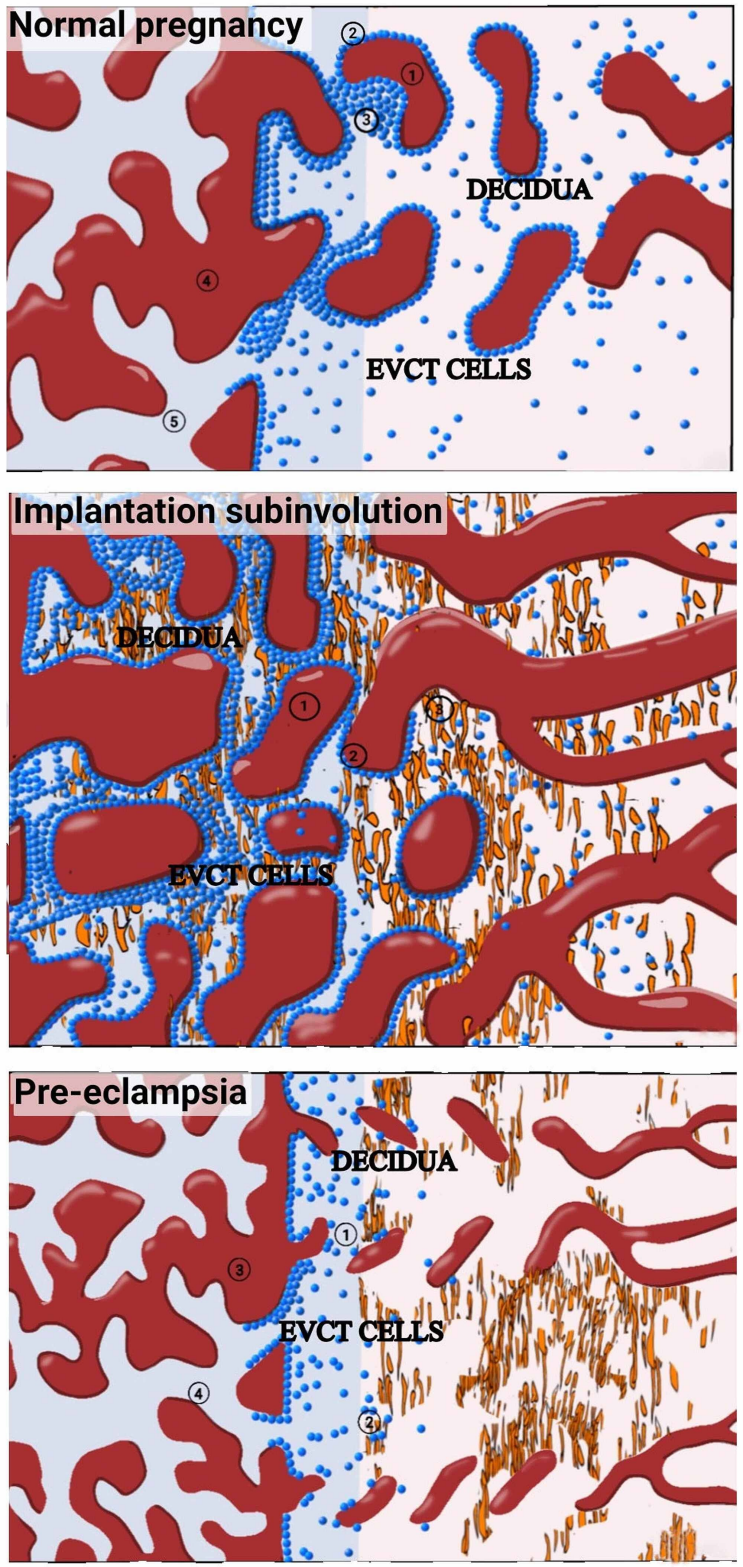
Pattern of trophoblastic invasion in normal pregnancy (top); pattern of trophoblastic persistence in uterine subinvolution (middle); and preeclampsia (bottom) 7A (top): pattern of trophoblastic invasion in normal pregnancy: 1. Normal pregnancy showing migration and invasion of extravillous endovascular and interstitial trophoblastic cells, resulting in large-caliber, high-flow but low-resistance vessels 2. Remodelled large-caliber, high-volume, and low-resistance vessels in pregnancy 3. Extravillous cytotrophoblastic cells (EVCTs) invade the interstitium and replace the endothelium becoming endovascular cytotrophoblastic cells. The EVCTs remodel the vessels to high-volume, high-caliber but low-resistance vessels 4. Interstitial extravillous trophoblastic cells 5. Fetal villi 7B (middle): pattern of trophoblastic persistence in uterine subinvolution - lobulated clusters of large patent subinvoluted uteroplacental arteries. The arteries show persistent endovascular extravillous cytotrophoblastic cells and interstitial cytotrophoblastic cells 1. Persistent low-resistance, dilated large-caliber vessels with increased blood flow in subinvolution 2. Persistent extravillous trophoblastic cells in the remodeled blood vessel wall, accounting for large caliber and low resistance of the vessel wall 3. Persistent interstitial extravillous trophoblastic cells, accounting for large caliber and low resistance of the vessel wall 7C (bottom): preeclampsia - poor investment and remodeling of the spiral arteries, resulting in low-caliber, high-resistance, and low-flow vessels 1 and 2. shallow and limited invasion of EVCT in the vessel wall. EVCT cells remain attached to anchoring villi. Endovascular invasion is absent with stiff spiral arterioles showing low caliber and high resistance 3. Fetal villi 4. Anchoring villi

Complex anatomic and physiologic changes occur in the uterine vessels at the time of pregnancy and during the postpartum period [15]. These changes are generally prominent at the placental implantation site. Early in pregnancy (6-10 weeks), placental-derived EVTs migrate in a retrograde manner and attack the decidual part of the maternal spiral arteries, thereby resulting in the development of endovascular “plugs” [4]. The musculoelastic medial tissues of the arteries are destroyed, whereas hyaline fibrinoid material is deposited. However, EVTs synchronously act to substitute the vascular endothelial layer and extravascular interstitial layer, leading to vasculature remodeling [9,11]. Following this, the endovascular EVTs also express angiogenic factors [9,11]. The overall result is vascular colonization, which spreads further to occupy a third of the myometrium [10,15]. The overall physiologic result of this colonization and remodeling activities is the development of large-caliber vessels characterized by high flow but low resistance, which are enough to meet the needs of the developing placenta and growing fetus [9,15] (Figure 7A).

Post-delivery, the physiologic mechanism of uteroplacental arterial involution is crucial for eliminating the remodeled changes in the vessel and for preventing major blood loss. Both the vascular and interstitial EVTs in the implantation site start to be replaced with maternal endothelial cells [6]. Several additional involutional changes occur during this process and include occlusive fibrointimal thickening, endarteritis, and thrombosis (Figures 1A, 1B). At the same time, the uterine smooth muscle contracts, leading to mechanical reduction and involution of the uterus and placental implantation site [7,9]. A major outcome of these changes at the implantation site is a decrease in blood loss [9]. Post-delivery, necrosis and sloughing of the decidua occur, together with the superficial endometrium, thereby completing the normal involution of the uterus [9].

Etiology of subinvolution

The normal remodeling of the vessels during the third trimester is either delayed or insufficient in the subinvolution of the placental implantation site; the uterus fails to return to the nonpregnant state [10], resulting in the persistence of low-resistance dilated vessels with the increased flow (Figure 7; Figures 2A, 2B, 2C).

Inadequate remodeling of the spiral arteries by EVTs is seen in patients with preeclampsia (Figure 7C); in subinvolution, the investment is substantial and the remodeling is persistent (Figure 7B). The respective pathophysiologic characteristics of subinvolution and preeclampsia could be an indication of the opposite ends of the spectrum of abnormal cellular interactions at the interface that occur between the fetal and maternal tissues (Figures 7A, 7B, 7C).

Another crucial piece of information about the mechanism of subinvolution is based on EVT apoptosis, i.e., EVTs seem to lose Bcl-2 expression in preeclampsia, whereas increased Bcl-2 expression has been described in subinvolution [6,9,15]. Persistent expression of this antiapoptotic protein perhaps helps maintain the uteroplacental vessels in a gestation-like condition when subinvolution occurs. However, in the case of long-lasting antiapoptotic gene expression, which supports cell survival [9,16], VSI develops in the previous site of placental implantation.

The molecular mechanisms include altered expression of the growth factors associated with the uterine scarring process and the upregulation of proangiogenic and antiangiogenic factors as well as receptors at the interface between the fetal and maternal tissues [4,6]. Investigators have also recognized genes that modify [6,10] the replacement of the decidua with recurrent endometrium, platelet aggregation, collagen deposition in vessels at the wounded site, and factor XIII activity [6].

VSI can also be enhanced by the occurrence of active acute inflammation, which can be enhanced via proinflammatory factor production and local tissue coagulopathy [6]. This acute situation becomes chronic with the formation of chronic disseminated intravascular coagulation [6] and predisposes women to secondary PPH [6,15].

Diagnosis of subinvolution

The Pathologist’s Perspective of a Case of PPH

Subinvolution can be recognized in severe cases by the characteristic clinical features and histologic findings in postpartum endometrial curettage samples from the placental site and hysterectomy specimens. However, ruling out the presence of chorionic villi, endometritis, placenta accreta, and gestational trophoblastic disease in this clinical setting is not sufficient.

In placental site subinvolution, the uterus is grossly enlarged and boggy. Multiple microscopic sections of the placental implantation site should be acquired to find the cause of hemorrhage and rule out other causes of secondary PPH [15]. Pathologic gross evaluation of the uterus reveals large ectatic-appearing vessels within the endomyometrium, which display scattered trophoblasts (EVT) cells within the walls with only minimal proliferation. The cytologic features of EVTs include a polygonal shape, abundant amphophilic cytoplasm, and vesicular nuclei [9]. Additionally, though less constant, microscopic features that have been described in subinvolution include the lack of internal elastic lamina duplication and partial or complete absence of a true endothelial lining.

The histologic findings in subinvolution probably reflect the nature of this process along a biologic continuum of changes, somewhere between appropriate involution and its complete absence. The extent of subinvolution varies from case to case; the diagnostic findings may be quantitatively limited in any given specimen [9,11]. In cases wherein the complete set of important findings is not observed, subinvolution may be proposed as a diagnostic possibility [9,15]. Therefore, the diagnosis of subinvolution should be only made in the correct clinical setting, which is secondary (delayed) PPH. Physiologic patency of uteroplacental arteries will be seen in the immediate (<24 hours) postpartum period [4,9] and should not be unmistakably interpreted as subinvolution [6].

An infrequent intravascular collection of obliterated fibrous chorionic villi can be identified; however, it should be properly investigated as to whether it accounts for the hemorrhage. Vascular subinvolution may be detected in the presence of rare degenerating intraluminal/intravascular chorionic villi, which is a valuable observation when the amount of villous tissue present does not account for the hemorrhage [9,15]. Therefore, the diagnosis of subinvolution should be carefully confirmed or ruled out in all cases, and clinicians should avoid the temptation to record only the presence or absence of villous tissues [9].

Sonographic Diagnosis

In establishing the sonographic diagnosis of the subinvolution of the placental site, it is necessary to visualize low-resistance vessels that are found along the inner third of the myometrium. In particular, gray-scale imaging is useful in revealing hypoechoic tortuous vessels found in the myometrium.

Another useful technique is pulsed-wave Doppler sonography, which can be used to confirm the vascular characteristics of an elevated optimum systolic velocity using a low-resistance waveform. Using this approach, the presence of increased areas of vascularity is an indication of the placental implantation site of either the second or third-trimester sonography [1]. It is important to note that lesions cannot be differentiated easily from congenital arteriovenous malformations. It is also extremely difficult to distinguish lesions from the acquired true arteriovenous malformations. However, arteriovenous malformations are still defined as high-flow uterine vascular malformations, rather than as subinvolution, which is defined by low-flow uterine vascular malformation [9].

In addition, the products of retained conception may mimic the above-mentioned findings and, therefore, should be documented as part of the differential diagnosis if echogenic tissue is observed in the endometrial cavity.

Management

The therapeutic management of secondary PPH is similar to that of primary PPH. The success of treatment requires coordination and collaboration of multidisciplinary care teams in order to achieve fast hemodynamic stabilization in the patient, replace the volume of the depleted blood, and prevent the occurrence of coagulopathy [6].

It is mandatory to perform a speculum examination of the lower genital tract and cervix to eliminate possible lacerations. Furthermore, it is compulsory to perform uterine ultrasonography to exclude the presence of retained placental tissue [6]. Vaginal bleeding should also be excluded in the differential diagnosis because of severe endometritis, gestational trophoblastic disease, or retained placental tissue. In this case, laboratory findings, such as elevated β-HCG levels, inflammatory markers, and positive blood and vaginal cultures, and expert ultrasound examination [6], are essential to help in excluding vaginal bleeding.

The symptomatic subinvolution of the placental site is a health condition of concern with bleeding, the management of which has remained a topic of debate. Some of the suggested techniques that can be used to manage conduction are vaginal packing, the use of uterotonic drugs, and blood and plasma unit transfusion. There are also more aggressive interventions that are considered standard therapies, such as uterine vessel ligation [1] and hysterectomy [1]. Furthermore, the treatment of subinvolution using percutaneous embolotherapy of the uterine arteries is supported by sufficient data. In such interventions, fertility can be retained following selective arterial embolization [9]. However, if percutaneous therapy fails to show positive outcomes, hysterectomy should be used as the final treatment option.

Clinical features and associations

Delayed PPH should be considered as an indication of subinvolution. Other signs of the condition are frequent bleeding that occurs between one week to several months post-parturition [9,16]. The onset of rising uterine bleeding is typically abrupt, thereby usually prompting the patients to seek immediate medical care [9].

## Conclusions

Placental site VSI is a rare form of secondary PPH that has always been underdiagnosed by clinicians. An immune-related cause involving miscommunication of the maternal and fetal tissues has been proposed regarding its occurrence. In the present study, the diagnosis was confirmed by the presence of endovascular EVTs and histology of the dilated as well as clustered myometrial arteries that were partly occluded by old and new thrombi. Nevertheless, confirmation was primarily obtained after conducting an emergency hysterectomy.

Furthermore, we considered that this kind of secondary PPH has an idiopathic cause and used the available data from a previous study to show that no predictive factors (e.g., features found on clinical examination with or without transabdominal ultrasonography) related to it are known. It is crucial to identify the severity of secondary PPH promptly to implement treatment via targeted therapy and in order to preserve fertility.
